# Biological brain aging, cognitive-motor decline and vascular risk: a multivariate imaging analysis of 40,579 individuals

**DOI:** 10.3389/fnagi.2026.1789408

**Published:** 2026-05-01

**Authors:** Marvin Petersen, Moritz A. Link, Carola Mayer, Felix L. Nägele, Maximilian Schell, Märit Jensen, Eckhard Schlemm, Jens Fiehler, Jürgen Gallinat, Simone Kühn, Raphael Twerenbold, Amir Omidvarnia, Felix Hoffstaedter, Kaustubh R. Patil, Simon B. Eickhoff, Götz Thomalla, Bastian Cheng

**Affiliations:** 1 Department of Neurology, University Medical Center Hamburg-Eppendorf, Hamburg, Germany; 2 Department of Diagnostic and Interventional Neuroradiology, University Medical Center Hamburg-Eppendorf, Hamburg, Germany; 3 Department of Psychiatry and Psychotherapy, University Medical Center Hamburg-Eppendorf, Hamburg, Germany; 4 Department of General and Interventional Cardiology, University Heart and Vascular Center, Hamburg, Germany; 5 Epidemiological Study Center, University Medical Center Hamburg-Eppendorf, Hamburg, Germany; 6 German Center for Cardiovascular Research (DZHK), Partner Site Hamburg/Kiel/Luebeck, Hamburg, Germany; 7 University Center of Cardiovascular Science, University Heart and Vascular Center, Hamburg, Germany; 8 Faculty of Medicine, Institute of Systems Neuroscience, Heinrich Heine University Düsseldorf, Düsseldorf, Germany; 9 Institute of Neuroscience and Medicine, Brain and Behaviour (INM-7), Research Center Jülich, Jülich, Germany

**Keywords:** brain age, cardiovascular risk, cerebral small vessel disease, cognitive function, motor function, neuroimaging, white matter hyperintensities

## Abstract

**Introduction:**

Age-related declines in cognitive and motor functions show highly variable trajectories. To better understand the underlying mechanisms, we investigated multivariate associative effects between modifiable vascular risk factors, biological brain aging, cognitive, and motor performance in 40,579 individuals from the population-based UK Biobank and Hamburg City Health Study.

**Methods:**

We employed partial least squares correlation analysis (PLS) to model associations between multi-domain cognitive and motor test scores and three distinct MRI-derived markers of biological brain aging: relative brain age (from morphometric brain imaging), white matter hyperintensity load, and peak width of skeletonized mean diffusivity. Furthermore, we conducted mediation analyses to assess if these markers mediate the impact of vascular risk on functional decline.

**Results:**

PLS identified a single dominant latent dimension explaining 94.7% of the shared variance between neuroimaging and behavior. This dimension linked higher biological brain aging markers – with relative brain age showing the strongest contribution – to poorer cognitive and motor performance, particularly in executive function and processing speed. Mediation analysis revealed that biological brain aging acts as a partial mediator for the negative effects of blood pressure, glucose, waist-hip ratio, and smoking load on cognitive and motor function. Notably, this mediating effect was not observed for cholesterol levels. These results were consistent across both cohorts.

**Discussion:**

Our study illustrates the associative interplay between vascular health, biological brain aging, and cognitive and motor performance, emphasizing the need for preventive strategies to maintain late-life independence in aging populations.

## 1 Introduction

Rising global life expectancy amplifies the challenge of age-related cognitive and motor impairment, threatening functional independence of individuals and burdening societies and healthcare systems worldwide (Crimmins, 2015). Although cognitive and motor abilities generally decline with age, there is substantial interindividual variability. While some individuals face cognitive impairment, dementia, and loss of independence, others maintain their cognitive and physical abilities well into advanced age (Krivanek et al., 2021). Unraveling the processes that uphold functional ability in older adults is essential for devising effective prevention and management strategies.

Mechanistic models have been proposed, suggesting that interindividual variability in mid and late life functionality arises from variations in biological brain aging, with some individuals exhibiting slower aging processes and others showing accelerated changes (Nyberg et al., 2012). While some contributors to these variations, such as genetic factors, are unmodifiable, others, including lifestyle and environmental influences, are modifiable. Here, cerebrovascular risk factors are considered to contribute to the variation in the rate of biological aging (de Lange et al., 2020).

Indicators of biological brain aging include changes in brain morphology, white matter microstructure, and presence of cerebral small vessel disease (CSVD) (Frey et al., 2021; Petersen et al., 2022b; Storsve et al., 2014, 2016). Magnetic resonance imaging (MRI) allows the characterization of these brain anatomical aspects in vivo. There are multiple neuroimaging markers theorized to capture variation in biological brain aging. These include (1) relative brain age, which measures the brain age gap – i.e., the discrepancy between chronological age and predicted biological age based on regional brain morphology (Franke and Gaser, 2019; Ning et al., 2020), (2) white matter hyperintensities of presumed vascular origin (WMH) indicative of CSVD (Frey et al., 2019; Hofmann et al., 2022), and (3) peak-width of skeletonized mean diffusivity (PSMD) reflecting global microstructural white matter integrity (Beaudet et al., 2020; Zanon Zotin et al., 2023).

Despite evidence linking these markers to cognition and motor function, a comprehensive understanding of the associations remains limited (Boyle et al., 2021; Jawinski et al., 2022; Richard et al., 2018). Much of the existing research has focused on individual imaging markers, vascular risk factors and cognitive or motor function scores without considering the potentially high covariance of the measures. Moreover, studies often have limited sample sizes, leading to inconsistent results, as emphasized by recent research highlighting the need for larger cohorts to establish reliable links between neuroimaging markers and behavior (Marek et al., 2022). Lastly, the role of vascular risk factors in biological brain aging, cognition and motor function is not fully understood, which is particularly relevant given their potential as intervention targets.

To bridge these gaps, we present a large-scale multi-modal neuroimaging analysis in two population-based studies, the UK Biobank (UKB) and the Hamburg City Health Study (HCHS). Combining key brain MRI markers of biological brain aging, vascular risk information, and comprehensive cognitive and motor phenotyping with multivariate, data-driven statistics – partial least squares correlation analysis (PLS) – we aimed to model the multivariate associative effects of biological brain aging, cognition, and motor function. Expanding on this, we investigated the role of biological brain aging in mediating the relationship between vascular risk, cognition and motor function in a mediation analysis. In sum, our analysis aimed to contribute to the understanding of the neurobiology underlying the decline in everyday cognitive and motor functioning to help identify potential diagnostic and treatment targets.

## 2 Materials and methods

### 2.1 Overview and study population

**FIGURE 1:**
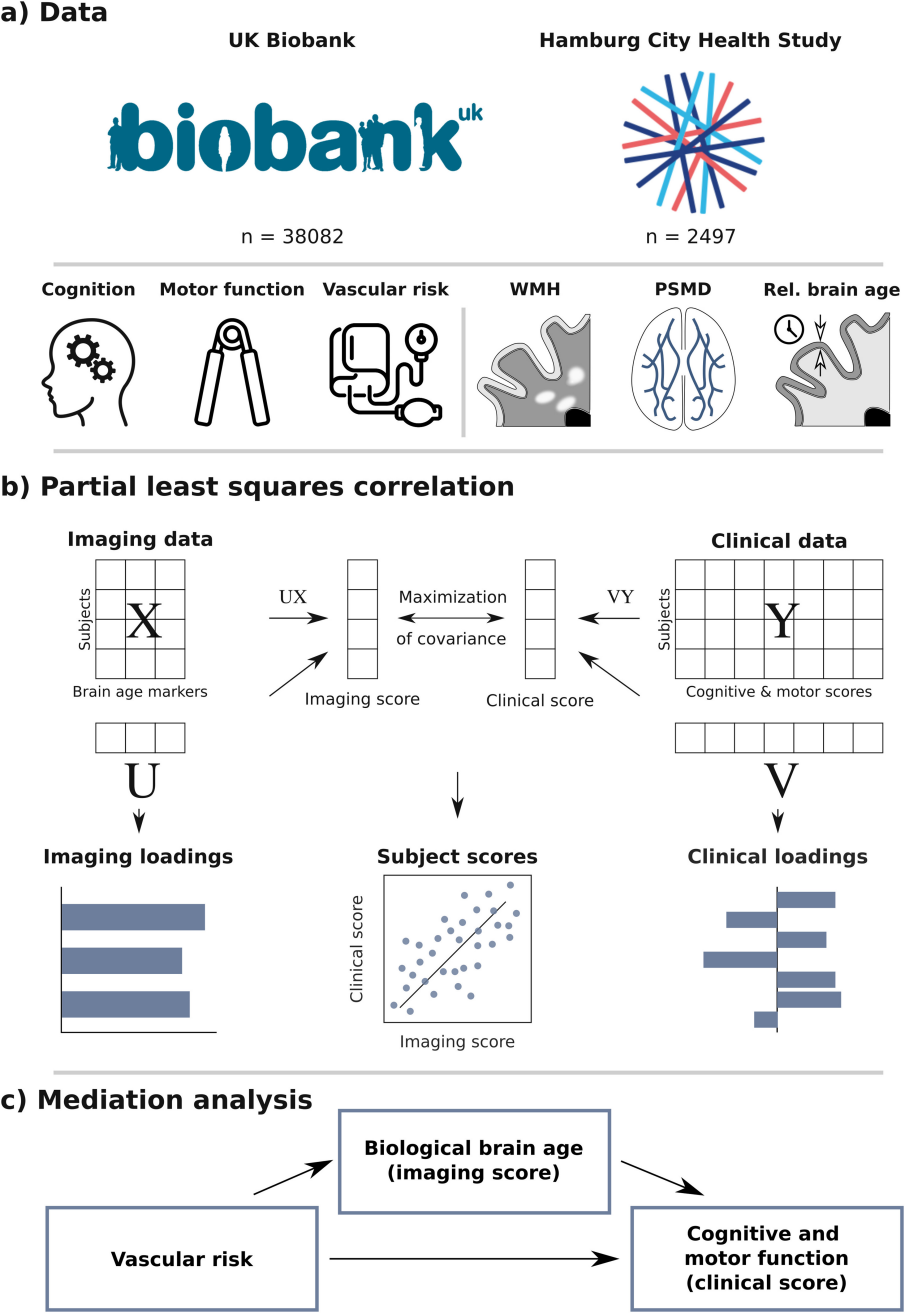
Methodology. **(a)** Population-based data from the UK Biobank and Hamburg City Health Study were used including cognitive test scores, motor performance scores, vascular risk measures and multimodal brain magnetic resonance imaging (MRI). Three different measures of biological brain aging were derived from anatomical and diffusion-weighted MRI: WMH, PSMD and relative brain age. **(b)** Imaging measures of biological brain age were related to cognitive and motor performance scores via partial least squares correlation analysis (PLS). PLS computes subject-specific scores (here imaging and clinical score), combining input data (X and Y) and respective loadings (U and V) through a linear combination. The loadings reveal the associative impact of the two input data domains, comparable to β-coefficients in linear regression. Together, subject-specific scores and loadings represent a latent variable. **(c)** The interplay between biological brain aging, vascular health, as well as cognitive and motor performance was investigated in a mediation analysis. We tested whether the relationship between different vascular risk measures and the clinical score – representing cognitive and motor performance, was statistically mediated by the imaging score – representing biological brain aging. PSMD, peak width of skeletonized of mean diffusivity; WMH, white matter hyperintensities of presumed vascular origin.

An overview of our methodology is provided in Figure 1. In brief, we utilized imaging and clinical data from the UKB and HCHS to investigate the complex relationships among biological brain aging, modifiable vascular risk, and cognitive and motor function. Following preprocessing, we derived relative brain age, WMH load, and PSMD from the imaging data. We then employed PLS to model the multivariate associations between these imaging markers and clinical scores of cognitive and motor function. Using the resulting subject-level PLS scores, which capture the associative effects between biological brain aging and clinical performance, we conducted a mediation analysis to test whether biological brain aging mediates the relationship between vascular risk measures and cognitive and motor test performance. All these analyses were performed separately in both the UKB (discovery cohort, *n* = 43,098) and HCHS (replication cohort, *n* = 2,652).

The UKB is an ongoing, multicenter, prospective population-based cohort study that recruited 500,000 individuals aged 40–69 across the United Kingdom. The study comprehensively collects genetic, physiological, lifestyle, environmental, and imaging data. As part of its multimodal imaging project, brain, heart, and abdomen scans are being acquired from a 100,000-participant subcohort. For this analysis, brain MRI data were available for 43,098 participants. In addition to UKB’s internal quality checks, further subject exclusion was performed using non-cancer illness codes^1^. This excluded individuals with conditions such as Alzheimer’s disease, alcohol/opioid/other addictions, amyotrophic lateral sclerosis, cerebral infarction, brain abscess, chronic neurological problems, encephalitis, epilepsy, hemorrhage, head injury, meningitis, multiple sclerosis, Parkinson’s disease, and skull fractures. Participants with PSMD values greater than three standard deviations from the mean were also removed. Ultimately, 38,082 subjects were included in the subsequent statistical analysis.

The HCHS is an ongoing, monocentric, prospective, population-based cohort study investigating the prevalence, risk, and prognostic factors of major chronic diseases (Jagodzinski et al., 2020). It is currently recruiting a random sample of 45,000 Hamburg residents aged 45–74. Participants undergo extensive baseline examinations, imaging, and genomic/proteomic characterization. At the time of this study, 2,652 MRIs were available from the initial 10,000 participants. HCHS subjects were matched to UKB criteria based on neuroradiological evaluation and self-reported diagnoses. Additionally, 155 HCHS subjects were excluded due to MRI acquisition and preprocessing issues. This resulted in a study cohort of 2,497 subjects for statistical analysis.

### 2.2 Ethics statement

The UKB’s ethical approval was granted by the North West Multi-Centre Research Ethics Committee (MREC). Details on the Ethics and Governance framework are provided online^2^ (Petersen et al., 2024b). The HCHS was approved by the local ethics committee of the Landesärztekammer Hamburg (State of Hamburg Chamber of Medical Practitioners, PV5131). Written informed consent was obtained from all participants. Data acquisition procedures followed the Good Clinical Practice (GCP) and Good Epidemiological Practice (GEP) guidelines according to the Declaration of Helsinki (Petersen et al., 2020).

### 2.3 Clinical data

Measurements of cognitive and motor function were investigated in this work. In brief, to facilitate interpretability and enhance comparability between examined cohorts, cognitive test results were harmonized (Coenen et al., 2023). First, cognitive test scores were assigned to cognitive domains. Within the cognitive domains, the corresponding tests were z-scored and averaged to obtain domain scores. If multiple tests were available for a specific cognitive domain, their z-scores were averaged. Trail Making Test and reaction time results were inverted and log transformed beforehand (Fawns-Ritchie and Deary, 2020). Furthermore, grip strength of the left and right hand was averaged. Mean hand grip strength was normalized based on division by the square of individual height following previous procedures (Nevill et al., 2022). For a comprehensive overview and detailed description of administered cognitive and motor tests and test domain assignment please refer to Supplementary Material 1.

### 2.4 Image processing

Magnetic resonance imaging acquisition protocols, preprocessing and quality assessment are documented in detail in Supplementary Material 2. Three measures of biological brain aging were derived from MR images: relative brain age, WMH load and PSMD. For the UKB, precomputed data were used, if possible. These included morphometric data computed via Freesurfer, WMH segmentations as well as preprocessed diffusion-weighted imaging data. HCHS data were fully processed by our team.

#### 2.4.1 Brain morphometry and relative brain age

Estimation of cortical and subcortical volumetric indices was performed leveraging Freesurfer (Dale et al., 1999; Fischl and Dale, 2000; Fischl et al., 2002). In brief, cortical thickness was measured on the vertex level as the distance between the pial surface and gray matter-white matter-boundary (Fischl and Dale, 2000). Cortical thickness and subcortical volumes were aggregated within regions of interest defined by the Desikan-Killiany cortical atlas and the aseg subcortical atlas (Desikan et al., 2006; Fischl et al., 2002). For the UKB, precomputed Freesurfer outputs were used, while HCHS data were processed by our team using an equivalent Freesurfer pipeline. Full details of acquisition parameters and preprocessing steps for both cohorts are provided in Supplementary Material 2.

Subsequently, region of interest-level morphological measures were used for relative brain age estimation (Bretzner et al., 2022; Ning et al., 2020). Notably, the estimation of relative brain age can be based on various imaging modalities. We chose to implement it using regional brain morphology, as this method is both common and computationally feasible for the scale of our study (More et al., 2023; Ning et al., 2020). Hence, the implemented measure captures the morphology of an individual brain in comparison to the population average: a positive relative brain age score represents advanced biological brain age, i.e., an “older appearing brain” compared to age-matched peers, whereas a negative relative brain age score indicates a relatively younger biological brain age (Smith et al., 2019). Importantly, relative brain age represents a variation of the commonly used brain age gap, i.e., the difference between chronological age and the predicted biological age from neuroimaging data (Franke and Gaser, 2019). Due to regression dilution bias, this original brain age gap measure can be negatively correlated with chronological age (Bretzner et al., 2022; Ning et al., 2020; Smith et al., 2019). Relative brain age addresses this ensuring orthogonality with chronological age (Bretzner et al., 2022; Ning et al., 2020). Computations were performed leveraging scikit-learn (v. 0.24.1) (Abraham et al., 2014). First, *predicted age* was obtained by fitting an ordinary least squares regression model with cortical thickness and subcortical volumetric indices as features and chronological age as the target. Fitting procedures were performed in a 5-fold cross-validation (see Supplementary Table 3 for prediction scores). In a second step, *expected age* was determined by fitting a separate linear model, but this time using chronological age as the input feature and *predicted age* as the target (Bretzner et al., 2022; Ning et al., 2020). Conceptually, expected age is the morphometry-predicted age typical for a given chronological age in our sample. Finally, relative brain age was calculated as the difference between predicted and expected brain age.


R⁢e⁢l⁢a⁢t⁢i⁢v⁢e⁢B⁢r⁢a⁢i⁢n⁢A⁢g⁢e=P⁢r⁢e⁢d⁢i⁢c⁢t⁢e⁢d⁢A⁢g⁢e−E⁢x⁢p⁢e⁢c⁢t⁢e⁢d⁢A⁢g⁢e

In accordance with previous procedures, predictions were performed separately for male and female participants (Sanford et al., 2022). For regression plots displaying orthogonality between relative brain age and chronological age see Supplementary Figure 4.

#### 2.4.2 White matter hyperintensity segmentation

White matter hyperintensity segmentation was performed by applying FSL’s Brain Intensity AbNormality Classification Algorithm (BIANCA) – a fully automated, supervised machine learning approach for WMH detection based on k-nearest neighbor classification – to FLAIR and T1w images (Griffanti et al., 2016). In the UKB, precomputed WMH segmentations were available, whereas HCHS WMH segmentations were generated by our team using the same BIANCA-based approach. WMH load was calculated as the ratio of WMH volume to intracranial volume computed via Freesurfer and logarithmized to ensure a normal distribution following previous procedures (Petersen et al., 2022a).

#### 2.4.3 Peak width of skeletonized mean diffusivity

Peak-width of skeletonized mean diffusivity was calculated via diffusion tensor imaging based on preprocessed diffusion-weighted images. First, diffusion tensors were modeled via a least-squares fit. In the UKB, preprocessed diffusion-weighted imaging data were used, while HCHS diffusion data were fully processed by our team. From the resulting tensors, mean diffusivity maps were derived. Skeletonized maps of mean diffusivity were obtained following the tract-based spatial statistics (TBSS) procedure (Smith et al., 2006). PSMD was calculated as the difference between the 95^th^ and 5^th^ percentiles of the MD voxel values within the skeleton (Petersen et al., 2022a).

### 2.5 Statistics

Visualization and statistical analysis were performed using Python (v. 3.9.7) leveraging matplotlib (v. 3.3.4), numpy (v. 1.2.1), pyls (v. 0.0.1), pandas (v. 1.2.4.), pingouin (v. 0.5.0), scikit-learn (v. 0.24.1), seaborn (v. 0.11.1), statsmodels (v. 0.13.1), confounds (v. 0.1.3), proplot (v. 0.9.5). Results were considered significant at a *p*-value of <0.05. To address multiple testing, reported *p*-values were false discovery rate-corrected (Benjamini and Hochberg, 1995). Descriptive statistics of the UKB and HCHS involved calculation of mean and standard deviations.

#### 2.5.1 Partial least square correlation analysis

Partial least squares correlation analysis was leveraged to investigate the multivariate associative effects between imaging and clinical markers. PLS handles multicollinearity among variables within each set, making it well-suited for analyzing highly correlated measures such as the different imaging markers of biological brain aging. Prior to PLS, imaging and clinical variables were residualized against chronological age, sex and education for deconfounding. This approach allows the PLS to identify associations between biological brain aging and physiocognitive performance that are independent of the linear effects of these demographic factors. Associations between imaging and clinical variables to age before residualization can be found in Supplementary Figures 5, 6. In a supplementary sensitivity analysis, intracranial volume (ICV) was additionally included as a confounder (Supplementary Figures 14, 24). PLS was performed via pyls^3^, which mirrors the PLS methodology originally detailed in McIntosh and Lobaugh (2004), McIntosh et al. (1996). For a detailed methodological description of PLS please refer to Supplementary Text 7 (Petersen et al., 2024b). In brief, PLS identifies associative effects between two sets of variables by identifying latent variables maximizing their covariance. In case of the presented study, the relationship of imaging markers of brain aging (relative brain age, WMH load, PSMD) and clinical data (cognitive function, motor test results) was modeled. Covariates (age, sex, and education) were included in the PLS analysis alongside the clinical variables to confirm they did not confound the modeled relationship. A latent variable consists of a singular value as well as loadings for both input domains, respectively quantifying the contribution of imaging and clinical variables to the overall covariance profile represented by the latent variable. In a simplified perspective, PLS can be considered as a dual regression resulting in interpretable coefficients for both multivariable data domains – i.e., a many-to-many mapping instead of a many-to-one mapping as provided by β coefficients in multiple regression. Significance testing of latent variables was performed by permuting subject labels of the imaging data domain and comparison of empirical singular values to the permuted distribution (n_*permutation*_ = 5,000). Stability of individual singular vector loadings was assessed via bootstrap resampling (n_*bootstrap*_ = 5,000). Bootstrapping involves random resampling with replacement, yielding a distribution of loadings for each variable. This enables for the computation of 95% confidence intervals for the clinical variables and for a bootstrap ratio ( = *singular vector weight*/*bootstrap*−*estimated standard error*) for the brain imaging markers. Furthermore, subject-level imaging and clinical PLS scores were calculated quantifying the extent an individual expressed the identified imaging or clinical covariance profile (Petersen et al., 2022b).

To enhance comparability with previous studies, we supplemented a linear regression analysis of the imaging and clinical variables associated in the PLS. Therefore, relative brain age, WMH load and PSMD were individually related to the cognitive and motor variables in linear regression analyses. All models were adjusted for chronological age, sex, and education. Effect sizes were reported as standardized β estimates.

Unlike relative brain age computations, WMH load and PSMD calculations are absolute measures and do not quantify deviations from the population mean. To investigate the stability of our results, we derived measures that quantify these deviations for WMH load and PSMD, aligning with the relative brain age concept. Essentially, we computed relative brain age measures but based on WMH load and PSMD instead of regional brain morphology to reflect deviations in CSVD burden and white matter microstructure. We then included these gap measures – termed WMH brain age and microstructural brain age – in place of the original absolute measures, alongside the initial relative brain age, in the PLS analysis.

#### 2.5.2 Mediation analysis

To disentangle the complex interplay between vascular risk factors, cognitive function, and motor performance, we performed a mediation analysis enabling the examination of biological brain aging as a potentially relevant intermediary in this link (Baron and Kenny, 1986). For this analysis, we used the subject-level imaging score and clinical score resulting from the PLS. These scores can be interpreted as summary measures like factors or principal components from other dimensionality reduction techniques: The subject-level imaging score represents a data-driven summary measure of biological brain aging markers while the subject-level clinical score summarizes cognitive and motor performance. The specific vascular risk factors examined as independent variables in separate mediation models were: systolic blood pressure, diastolic blood pressure, total cholesterol, HDL cholesterol, LDL cholesterol, triglycerides, glucose, waist-hip ratio, and pack years. Pack years were defined as the number of cigarette packs smoked per day multiplied by the number of years of smoking. We tested the mediating effect of the subject-level imaging score on the association of vascular risk factors and the subject-level clinical score. A mediation analysis decomposes the total effect of the vascular risk factors on the subject-level clinical score into two components: (1) the direct, i.e., non-mediated, effect of vascular risk on the clinical score, and (2) the indirect effect, i.e., the proportion of the effect that can be attributed to the subject-level imaging score. An indirect effect was considered to mediate the relationship between vascular risk and clinical performance when a vascular risk factor was significantly associated with the mediator, the mediator was significantly associated with the subject-level clinical score and the link between a vascular risk factor and the subject-level clinical score was reduced (partial mediation) or became non-significant (full mediation) when controlling for the mediator. The presence of a significant mediating effect was determined using bootstrapping (n_*bootstrap*_ = 5,000). Models were adjusted for chronological age, sex, and education. Input variables to the mediation analysis were z-scored beforehand, so standardized effect measures are reported.

## 3 Results

### 3.1 Descriptive statistics of UKB and HCHS

Application of exclusion criteria and quality assessment resulted in the exclusion of *n* = 5,016 subjects in the UKB sample and *n* = 155 subjects in the HCHS sample. The final analysis sample consisted of *n* = 38,082 UKB subjects and *n* = 2,497 HCHS subjects (Supplementary Figure 8). Descriptive statistics are displayed in Table 1. Supplementary Figure 9 shows statistical comparisons of overlapping variables between the two cohorts. While the two cohorts were broadly comparable in age and key vascular risk factors, statistically significant differences were observed for several variables, including sex distribution, education, diastolic blood pressure, HDL cholesterol, and WMH load (Supplementary Figure 9).

**TABLE 1:** Descriptive statistics.

Demographics and vascular risk factors	UKB (*n* = 38,082)^a^	HCHS (*n* = 2,497)^a^
Age, years	63.7 ± 7.6	63.79 ± 8.3
Sex, % female	50.2	44.13
Education, ISCED	4.5 ± 1.3	2.43 ± 0.6
Pack years	18.8 ± 15.7	8.09 ± 17.4
Waist-hip ratio	0.9 ± 0.1	0.94 ± 0.09
BP_*systolic*_, mmHg	138.6 ± 18.6	141.41 ± 19.5
BP_*diastolic*_, mmHg	78.7 ± 10	82.64 ± 10.1
Cholesterol, mmol/l	5.7 ± 1.1	5.43 ± 1.1
LDL, mmol/l	3.6 ± 0.8	3.15 ± 1
HDL, mmol/l	1.5 ± 0.4	1.66 ± 0.5
Triglycerides, mmol/l	1.6 ± 1	1.36 ± 0.8
Glucose, mmol/l	5.0 ± 1	5.38 ± 1.1
Imaging markers		
Relative brain age	−0.01 ± 3.8	−0.001 ± 4.6
PSMD, 10^–4^ mm^2^/s	2.29 ± 0.4	2.26 ± 0.3
WMH load, %	0.29 ± 0.4	0.17 ± 0.2
WMH volume, ml	4.63 ± 5.5	2.56 ± 3.2
Cognitive tests		
Numeric Memory Test	6.79 ± 1.3	–
Trail Making Test A, s	22.45 ± 8.1	39.92 ± 14.3
Trail Making Test B, s	57.13 ± 25.7	89.26 ± 38.3
Matrix Pattern Completion Test	8.03 ± 2.1	–
Fluid intelligence	6.66 ± 2.1	–
Reaction time, s	0.59 ± 0.1	–
Paired Associate Learning Test	6.98 ± 2.6	–
Tower Rearranging Test	9.97 ± 3.2	–
Symbol Digit Substitution Test	19.06 ± 5.2	–
Word List Recall Test	–	7.77 ± 1.8
Multiple Choice Vocabulary Intelligence Test B	–	31.26 ± 3.6
Animal Naming Test	–	24.85 ± 6.9
Motor tests		
Mean hand grip strength, kg	30.3 ± 10.3	34.26 ± 10.6
Average acceleration, milli-gravity	28.5 ± 7.8	–
Timed Up And Go Test (s)	–	7.02 ± 1.7

ISCED, International Standard Classification of Education; kg, kilogram; l, liter, mm, millimeters; mmHg, millimeter mercury; s, seconds.

*^a^*Presented as mean ± standard deviation.

### 3.2 Imaging markers of biological brain aging are associated with cognitive and motor function

In the following, we report results for the UKB. For details on HCHS results refer to the Supplementary materials.

**FIGURE 2:**
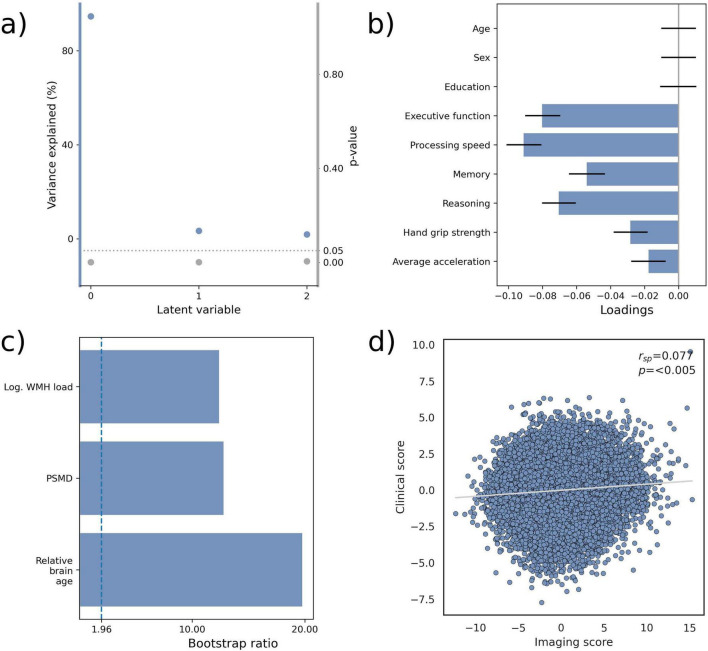
Partial least squares correlation results. **(a)** Overview of detected latent variables with the first latent variable (labeled as 0) explaining 94.7% of observed shared variance. Blue dots indicate the values of the observed shared variance, while gray dots indicate the false discovery rate-corrected *p*-value (P_*FDR*_). **(b)** Loadings of the clinical variable set consisting of cognitive and motor test results. Error bars indicate the 95% confidence interval obtained by bootstrap resampling. **(c)** Bootstrap ratios of the imaging variable set. The vertical dashed line represents the significance threshold (bootstrap ratio > 1.96). **(d)** Relationship of subject-level imaging and clinical scores. r_*sp*_, Spearman correlation; Log WMH load, logarithmized white matter hyperintensity load; PSMD, peak width of skeletonized mean diffusivity.

Partial least squares correlation analysis revealed three significant latent variables (Figure 2a) each representing a many-to-many mapping relating imaging and clinical markers. The first latent variable accounted for 94.7 % of shared variance and was thus further examined. Specifically, the first latent variable corresponded with a clinical covariance pattern of significantly worse cognitive and motor performance across all considered clinical variables (0 ∉ [95% confidence interval]; Figure 2b and Supplementary Table 10). Notably, cognitive domain scores of executive function and processing speed showed the strongest contribution to the covariance profile as indicated by the highest loading to the latent variable. Chronological age, sex and education did not significantly contribute to the covariance pattern (0 ∈ [95% confidence interval]) indicating sufficient effects of deconfounding. Regarding the imaging markers of biological brain aging, relative brain age ([bootstrap ratio], 19.8), PSMD (12.8) and WMH load (12.4) exhibited a significant positive (>1.96) contribution to the covariance pattern (Figure 2c). Therefore, a higher relative brain age, WMH load and PSMD corresponded with worse cognitive and motor performance. Of note, relative brain age contributed most strongly among investigated imaging markers as indicated by the highest bootstrap ratio. Subject-specific imaging and clinical scores were calculated and showed a positive correlation (*r*_*sp*_ 0.077, *p* < 0.05, Figure 2d), indicating that individuals expressing, to a greater extent, the clinical covariance pattern (worse cognitive and motor performance) also express, to a greater extent, the imaging pattern (higher relative brain age, WMH load, PSMD). This relationship was stable across a 10-fold cross-validation (avg. *r*_*sp*_ = 0.096 Supplementary Table 11). The PLS results remained stable when incorporating brain age gap measures based on WMH load and PSMD alongside relative brain age (Supplementary Figure 12), when including individual cognitive scores instead of domain scores (Supplementary Figure 13) or when including intracranial volume as an additional confounder (Supplementary Figure 14).

In addition, we performed multiple linear regression analyses between individual imaging markers of biological brain aging as well as cognitive and motor performances confirming the associations suggested by the PLS (Supplementary Materials 15, 16). Cross correlation matrices of imaging and clinical markers are shown in Supplementary Materials 17, 18. The abovementioned results were reproducible in the HCHS sample (Supplementary Materials 19–28).

### 3.3 Mediation analysis

**FIGURE 3:**
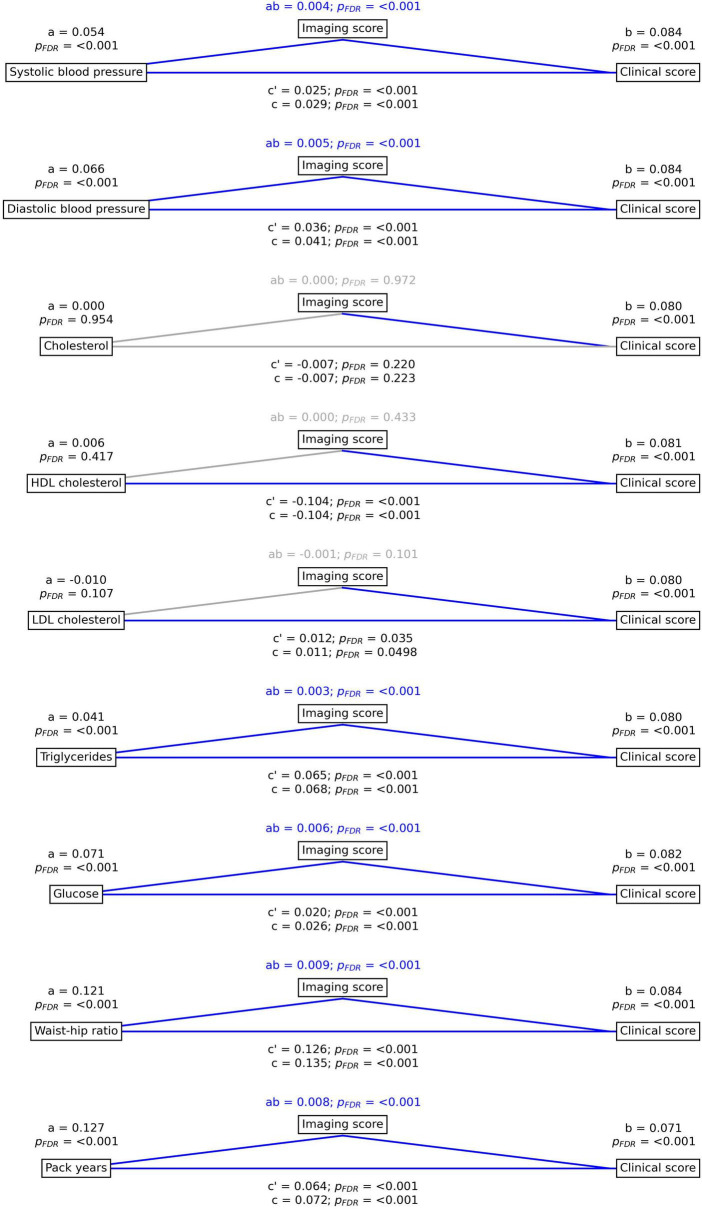
Mediation analysis results. Mediation effects of subject-level imaging score on the relationship between vascular risk factors and subject-level clinical scores summarizing cognitive and motor performance. Path plots display standardized effects and *p*-values: **(a)** vascular risk factor to subject-level imaging score, **(b)** subject-level imaging score to clinical score, (ab) indirect effect (c’) direct effect and **(c)** total effect. Significant paths are highlighted in blue; non-significant in light gray. If the indirect path was significant the text for ab is highlighted in blue. p_*FDR*_, false discovery rate-corrected *p*-value.

We observed significant total effects (as indicated by “c,” in Figure 3) between various vascular risk factors and the subject-level clinical score, which summarizes overall cognitive and motor performance. Specifically, higher systolic blood pressure (c = 0.029, *p*_*FDR*_ < 0.001), diastolic blood pressure (c = 0.041, *p*_*FDR*_ < 0.001), LDL cholesterol (c = 0.011, *p*_*FDR*_ = 0.0498), triglycerides (c = 0.068, *p*_*FDR*_ < 0.001), glucose (c = 0.026, *p*_*FDR*_ < 0.001), waist-hip ratio (c = 0.135, *p*_*FDR*_ < 0.001), pack years (c = 0.072, *p*_*FDR*_ < 0.001) and lower HDL cholesterol (c = –0.104, *p*_*FDR*_ < 0.001) were directly linked to poorer clinical scores.

Mediation analysis results further confirmed that subject-level imaging scores partially mediate the link between vascular risk factors and clinical scores (Figure 3). This held true for systolic blood pressure (ab = 0.004, *p*_*FDR*_ < 0.001; c’ = 0.025, *p*_*FDR*_ < 0.001), diastolic blood pressure (ab = 0.005, *p*_*FDR*_ < 0.001; c’ = 0.036, *p*_*FDR*_ < 0.001), triglycerides (ab = 0.003, *p*_*FDR*_ < 0.001; c’ = 0.065, *p*_*FDR*_ < 0.001), glucose (ab = 0.006, *p*_*FDR*_ < 0.001; c’ = 0.020, *p*_*FDR*_ < 0.001), waist-hip ratio (ab = 0.009, *p*_*FDR*_ < 0.001; c’ = 0.126, *p*_*FDR*_ < 0.001) and pack years (ab = 0.008, *p*_*FDR*_ < 0.001; c’ = 0.064, *p*_*FDR*_ < 0.001). While these indirect effects (ab) were statistically significant, indicating a mediating role of the imaging score, their magnitudes relative to the total effects (c) were generally small (e.g., for systolic blood pressure, the mediated effect ab = 0.004 constituted approximately 13.8% of the total effect c = 0.029). The links between the subject-level cholesterol, HDL cholesterol, LDL cholesterol and the clinical score were not significantly mediated. These results were reproducible in the HCHS except for systolic and diastolic blood pressure showing no significant link to the subject-level clinical score (Supplementary Figure 29).

## 4 Discussion

Understanding individual differences in aging trajectories is vital for informing strategies to maintain physical and cognitive health in mid and later life. In this work, we linked structural neuroimaging markers of biological brain aging with cognitive and motor test performances in two population-based samples with a total of 40,579 individuals. We report on three main findings: (1) multivariate, data-driven statistics revealed a subtle but consistent link between advanced biological brain aging as well as lower cognitive and motor performance independent of chronological age, sex and education; (2) vascular risk factors were significantly linked to PLS-derived aggregate measures of both biological brain aging as well as cognitive and motor performance; (3) biological brain aging mediated the link between the vascular risk factors and cognitive as well as motor performance. These results applied to both investigated subcohorts: the UKB (discovery cohort) and the HCHS (replication cohort). In essence, our study demonstrates a clear link between biological brain aging and cognitive and motor abilities. In this context, biological brain aging acts as a partial mediator between common vascular risk factors and impaired cognition and motor function.

### 4.1 PLS reveals a latent dimension integrating biological brain aging, cognition and motor function

Partial least squares correlation analysis identified a single dominant latent variable associating higher markers of biological brain aging with lower cognitive and motor scores. This variable explained 94.7% of the shared variance between imaging and clinical data, indicating a largely monodimensional relationship between brain aging and physiocognitive outcomes. Relative brain age, WMH load, and PSMD all contributed significantly to this latent variable, highlighting substantial overlap among these diverse imaging markers in their association with cognitive and motor function. This convergence aligns with previous work showing high covariance among the examined imaging markers as well as among cognitive and motor test performances (Busby et al., 2022; Hofmann et al., 2022; Lee et al., 2022).

Among the markers, relative brain age exhibited the largest contribution (highest bootstrap ratio in UKB and HCHS). While speculative, this pattern suggests that, beyond shared variance, these markers may capture complementary or partly distinct pathophysiological processes. The stronger influence of relative brain age may reflect global morphological changes encompassing broader age-related neural processes – such as neuronal or synaptic loss or early subclinical neurodegeneration – beyond the white matter alterations and small vessel disease captured by PSMD and WMH load. Consequently, combined cortical and subcortical morphology, as indexed by relative brain age, may provide a more direct measure of “biological brain age” and its functional relevance.

Although the correlation between individual imaging and clinical scores was statistically significant, it was small in magnitude (ρ = 0.077 in UKB; ρ = 0.085 in HCHS). Such subtle effect sizes are typical in large-scale population neuroscience, where reliable brain–behavior associations are often small (Marek et al., 2022). The replication of these associations across two independent cohorts, however, underscores that the link between biological brain aging and cognitive–motor function, while small, is reproducible.

### 4.2 Biological brain aging is associated to worse cognitive performance across domains

Previous studies have individually connected single imaging markers of biological brain aging such as brain age gap, WMH, or PSMD with cognitive performance, yet the involvement of all cognitive domains is uncertain due to inconsistent findings (Boyle et al., 2021; Deary et al., 2019; Prins and Scheltens, 2015). Our results from PLS and multiple linear regression underscore that associations between brain aging and cognition are not confined to specific cognitive domains, a result likely attributable to our study’s ability to reliably detect subtle effects due to a large sample size. This aligns with previous research showing widespread influence of WMH and brain age gap measures across key cognitive areas (Hamilton et al., 2021; Jawinski et al., 2022). An alternative hypothesis could be that the observed covariance in domain-specific cognitive tests stems from their shared reliance on certain cognitive functions, such as memory tests also tapping into attention and executive function. Importantly, while all cognitive domains were found to be associated with brain aging, our findings highlight executive function and processing speed as the most strongly affected areas, consistent with existing literature linking age-related brain changes to declines in these faculties (Heckner et al., 2021; Petersen et al., 2022b). Furthermore, these results corroborate past studies connecting WMH and PSMD to specific impairments in executive function and processing speed, characteristic of cerebral small vessel disease (Deary et al., 2019; Low et al., 2020).

### 4.3 Imaging markers of brain aging link to motor performance

Turning to motor function, we could show that imaging markers of biological brain aging link to motor performance, notably hand grip strength and physical activity measured via accelerometry, though the correlation was weaker than with cognitive function. The association between biological brain aging markers and motor function is less documented than that with cognitive measures; however, this link is supported by the known relationship between motor skills and cognition in mid to later life indicating shared variance (Duchowny et al., 2022). Analyzing cognitive and motor functions jointly in a multivariate framework like PLS allowed us to identify common underlying pathways of age-related decline, reflecting their established co-vulnerability and shared neural substrates in aging. This integrated approach provides a more comprehensive understanding of overall “physiocognitive” independence in older adults. Our findings align with previous studies demonstrating a negative correlation between brain aging and motor performance, specifically with hand grip strength and physical activity (Cole et al., 2018; Fleischman et al., 2015). Pathomechanistically, older adults engage a more widespread network of brain areas for motor control, particularly the prefrontal cortex and basal ganglia, which are highly susceptible to aging (Raz et al., 1997). This could lead to a mismatch in neural resource allocation. Moreover, decreased physical activity may both result from and contribute to biological brain aging. Given the neuroprotective benefits of physical exercise, a lack of it might increase the risk for onset and progression of neurodegenerative processes, highlighting an opportunity for targeted interventions (Ahlskog et al., 2011).

### 4.4 Biological brain aging mediates the link between vascular risk, cognition and motor function

Vascular risk is a key modifiable factor influencing brain structure and function, with higher risk associated with poorer structural integrity and reduced cognitive performance (Borshchev et al., 2019; Petersen et al., 2024b). Our mediation analysis revealed significant associations between vascular risk factors and clinical PLS scores, which reflect adherence to the identified clinical covariance pattern – essentially, worse cognitive and motor performance. Importantly, this relationship was partially mediated by the imaging PLS score, a measure of biological brain aging. This finding suggests that variations in macrostructural and microstructural brain integrity help explain how vascular risk translates into functional decline, highlighting the mechanistic role of brain aging in the clinical consequences of cerebrovascular disease (Petersen et al., 2024a,b).

Although these indirect effects were small compared to the total effects, their presence underscores that brain aging markers capture a biologically plausible pathway linking vascular risk to cognitive and motor deficits. At the same time, the relatively small effect sizes indicate that additional factors – such as microvascular changes, inflammation, or lifestyle influences – likely contribute substantially to these relationships.

Overall, our results contribute to insights on the connections between vascular risk and both structural and functional aspects of brain health, suggesting potential clinical applications. Interventions targeting vascular risk – through prevention or treatment – may slow biological brain aging, thereby mitigating cognitive and motor decline. Future strategies could leverage brain imaging to personalize therapies and identify individuals most likely to benefit from such interventions.

## 5 Strengths and limitations

Strengths of this work lie in its large sample size, which minimizes the overestimation of effects and enhances reproducibility (Marek et al., 2022). Further strengths include the replication of findings in an independent sample, advanced neuroimaging and statistical techniques, and comprehensive cognitive phenotyping. A novel aspect of this study is its integrated, large-scale examination of the relationships between structural brain changes and both cognitive and physical performance. Although prior research has investigated these associations separately, they have not been unified within a single comprehensive framework. This approach offers new insights into their interdependence and enables direct comparison of these factors within a shared analytical context. However, several limitations must be acknowledged. First, the cross-sectional design precludes causal inference. Second, although harmonized, the specific cognitive and motor assessments differed between UKB and HCHS, which may complicate direct comparison of test scores. Third, our cohorts primarily consisted of middle-aged to older adults of European descent, limiting generalizability to younger or more diverse populations. Fourth, the effect sizes observed were statistically significant but small in magnitude. While such small effects are expected in large-scale population neuroscience, and may still be clinically meaningful at a population level, their immediate relevance for individual-level prediction or diagnosis is limited. Finally, although our mediation analyses indicated a mediating role of biological brain aging, the direct effects of vascular risk factors remained dominant, suggesting that brain aging represents only one of multiple mechanisms underlying cognitive and motor decline.

## 6 Conclusion

In summary, our findings suggest a primarily low-dimensional relationship between biological brain aging and cognitive and motor performance. Our results highlight the role of vascular risk factors in contributing to accelerated brain aging and worse cognitive and motor performance, advocating for the implementation of effective preventive strategies for upholding functional independence up until higher age.

## Data Availability

UK Biobank data can be obtained via its standardized data access procedure (https://www.ukbiobank.ac.uk/). HCHS participant data used in this analysis is not publicly available for privacy reasons, but access can be established via request to the HCHS steering committee.

## Footnotes

1
https://biobank.ndph.ox.ac.uk/showcase/coding.cgi?id=6

2
https://www.ukbiobank.ac.uk/media/0xsbmfmw/egf.pdf

3
https://github.com/rmarkello/pyls
